# The Comorbidity of AD and PD: Exploring Clinical, Pathological, and Biomarker Interactions

**DOI:** 10.14336/AD.2025.0301

**Published:** 2026-06-13

**Authors:** Qi-Lei Zhang, Yu Liu, Tian Tu, Xiao-Xin Yan

**Affiliations:** ^1^Department of Anatomy and Neurobiology, Central South University Xiangya Basic Medical School, Changsha, Hunan 410013, China.; ^2^Department of Neurology, Xiangya Hospital, Central South University, Changsha, Hunan 410008, China.

**Keywords:** Comorbidity, Clinical Manifestations, Spatiotemporal Evolution, Propagation and Interaction of Pathological Biomarker

## Abstract

Alzheimer’s disease (AD) and Parkinson’s disease (PD) are the most common neurodegenerative disorders, primarily characterized by cognitive decline and motor dysfunction, respectively. The comorbidity of AD and PD further increases disease complexity and presents significant challenges for clinical diagnosis and treatment. Due to substantial clinical overlaps in the advanced stage of AD and PD, comorbid cases are frequently misdiagnosed as a single disease, which hinders early recognition and timely intervention. Based on pathological observations from patients with comorbidity of AD and PD, this review discusses the prevalence and clinical features of AD-PD comorbidity, the spatiotemporal progression and potential interactions of key pathological proteins, including β-amyloid (Aβ), phosphorylated tau (pTau), and α-synuclein (α-syn). The aim of this review is to update potential new insights and strategies to improve diagnostic accuracy, advance personalized therapeutic approaches, and guide future research into the underlying mechanisms of comorbidity.

## 1. Introduction

Cognitive decline, memory loss, and behavioral changes are the major classical clinical manifestations of Alzheimer’s disease (AD) [1]. In the advanced stages of AD, patients may experience motor dysfunction, including rigidity, bradykinesia, and gait impairment [2, 3]. Parkinson disease (PD) on the other hand is primarily characterized by motor dysfunction, while approximately 20% to 40% of advanced-stage PD patients also have cognitive impairment or dementia [4, 5]. While AD and PD are generally recognized as distinct disease entities, they share overlapping clinical features, making it extremely challenging to differentiate between late-stage AD, late-stage PD, and their comorbidities. As a result, the comorbidities are often overlooked. Furthermore, the presence of comorbidities may accelerate the progression of both diseases, resulting in poorer brain dysfunction and quality of life with a worse prognosis for the patient [6, 7].

**Figure 1.**
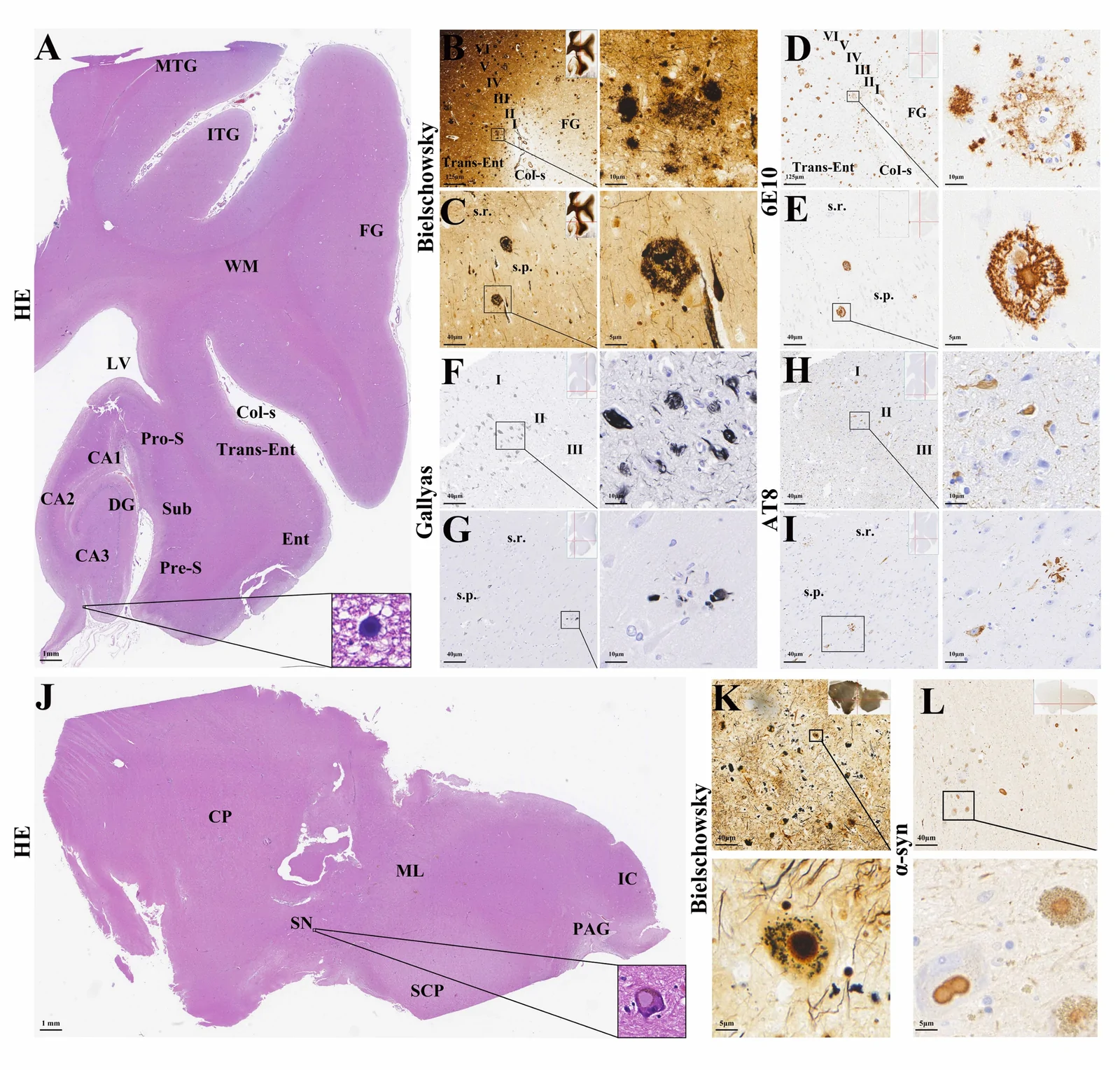
**Histopathological and immunohistochemical staining at the hippocampal and substantia nigra levels**. (**A**) HE stain of the hippocampus with magnified inset showing corpora amylacea. (**B**) Bielschowsky silver stain of the middle temporal gyrus (MTG), with a magnified view of diffuse amyloid plaques. (**C**) Bielschowsky silver stain of the hippocampus, showing a typical cored compact amyloid plaque. (**D**) 6E10 immunohistochemistry of the MTG, highlighting diffuse plaques in the enlarged inset. (**E**) 6E10 immunolabeling in the hippocampus, with a cored amyloid plaque shown in the magnified view. (**F**) Gallyas silver stain of the entorhinal cortex (Ent) shows numerous mature-looking and ghost tangles as shown in the inset. (**G**) Gallyas silver stain of the hippocampal CA1 region shows many magnified tangles. (**H**) AT8 immunohistochemical stain of the entorhinal cortex shows many tau-positive tangles. (**I**) The AT8 immunolabeling of the hippocampal CA1 region shows labeled neuronal somata and neuritic profiles in the inset. (**J**) The HE stain of the substantia nigra (SN) shows corpora amylacea. (**K**) Lewy bodies within the SN in the Bielschowsky silver stain. (**L**) α-Synuclein immunohistochemical stain of the SN shows a Lewy body and neurites in the magnified inset. Scale bars are indicated in each panel. Abbreviations: MTG: Middle Temporal Gyrus, ITG: Inferior Temporal Gyrus, FG: Fusiform Gyrus, WM: White Matter, LV: Lateral Ventricle, pro-S: Prosubiculum, Col-S: Collateral Sulcus, Trans-Ent: Transentorhinal Cortex, Ent: Entorhinal Cortex, CA1/CA2/CA3: Cornu Ammonis regions 1, 2, 3, DG: Dentate Gyrus, Sub: Subiculum, Pre-S: Presubiculum, I-VI: Cortical layers I to VI, s.r.: Stratum Radiatum, s.p.: Stratum Pyramidale, CP: Cerebral Peduncle, SN: Substantia Nigra, ML: Medial Lemniscus, SCP: Superior Cerebellar Peduncle, PAG: Periaqueductal Gray, IC: Inferior Colliculus.

The absence of explicit diagnostic criteria for these comorbid cases contributes to a significant mismatch between antemortem clinical diagnosis and postmortem pathological diagnosis. A significant number of patients were diagnosed with AD or PD during life, and upon postmortem examination they were found to contain multiple neuropathological changes, including Aβ plaques, pTau tangles, as well as Lewy bodies. This suggests that the possibility of comorbidity may be overlooked in clinical assessments [8, 9]. Substantial progress has been made over the past 10 years in human brain banking in China, with a network of brain banks established among major medical universities via the willed body-donation programs [10]. Postmortem brains are pathologically evaluated following a standardized protocol. This involves an extensive neuropathological assessment, which includes testing for Aβ, pTau and α-synuclein [11]. We have assessed a significant number of cases within the Xiangya brain bank and observed the coexistence of pathological markers associated with both AD and PD, yet the clinical diagnosis typically considers only a single disease entity or even neither is made antemortem. An example of this is seen in a 70-year-old male patient who was clinically diagnosed with PD, but postmortem neuropathological examination revealed mixed pathological features. These features included numerous diffuse and compact Aβ plaques in the neocortex, limbic system, and midbrain. Significant neurofibrillary tangles in the entorhinal cortex and hippocampus, and prominent Lewy bodies in the midbrain, limbic system, and in a number of cortical regions. Figure 1 illustrates the pathological features observed in the hippocampus and substantia nigra in this case. Even in the Hematoxylin-Eosin (HE) stained sections of the hippocampal we observe abundant corpora amylacea (amyloid bodies) both in hippocampal and subpial areas (Fig. 1A, 1J and enlarged image). Bielschowsky silver stain and Aβ (clone 6E10) immunohistochemistry (IHC) show both compact and diffuse plaques in the temporal lobe (Fig. 1B, D and enlarged image), but in the hippocampus the compact plaques are more predominant (Fig. 1C, E and enlarged image). Gallyas silver and AT8 immunohistochemical staining confirm abundant presence of ghost tangles in the entorhinal cortex (Fig. 1F, H and enlarged image), as well as neuritic plaques (NPs) and mature neurofibrillary tangles in the CA1 region of the hippocampus (Fig. 1G, I and enlarged image). Additionally, numerous Lewy bodies are found in HE stain of the midbrain sections (Fig. 1J and enlarged image), which are also seen in Bielschowsky silver stain and α-synuclein IHC (Fig. 1K, L and enlarged image).

The mismatch between clinical diagnosis during the patient's life and the pathological findings found at postmortem might account for the limited effectiveness of treatments. Treatment regimens targeting only one disease entity are often insufficient to improve the clinical outcome of patients with comorbidities ^[12]^. On the other hand, combining several therapeutic agents intended for use in either AD or PD may interact with one another and potentially lead to serious side effects that could diminish the overall effectiveness of individual treatments when applied to patients with comorbid conditions [13, 14]. We discuss a number of issues regarding the comorbidity of AD and PD, including epidemiological features, clinical manifestations, spatiotemporal evolution of disease lesions and propagative patterns, and the potential interaction mechanisms of comorbidities.

## 2. The Epidemiology of Comorbid in AD and PD

Both AD and PD are prevalent in the aging populations [15-17]. Thus, age is a significant determinant of AD and PD prevalence, with the percentage of individuals diagnosed with AD-type dementia and PD increasing as age advances. A recent study reported that 5% of people aged 65 to 74, 13.1% of people aged 75 to 84, and 33.3% of those aged 85 or older are affected by AD [18]. Like AD, the number of PD patients aged over 80 is 2.6-fold higher than those aged 60-69 [19].

AD and PD co-occurrence complicates disease progression, diagnosis and treatment. The lack of clear diagnostic standards leads to substantial variations in the reported prevalence of comorbidities across different studies. One study reported that the prevalence of parkinsonism in AD patients ranges between 15% and 45% [20, 21]. Another study involving 1,500 patients with confirmed AD found that 18% also exhibited clinical signs of parkinsonism [5]. In contrast, a larger cohort study reported that up to 30-40% of Alzheimer’s patients also displayed significant parkinsonian features, such as tremors, rigidity, and bradykinesia [20]. Similarly, the prevalence of AD-type dementia in PD patients is notable. It has been estimated that up to 80% of PD patients will develop some form of cognitive impairment, with approximately 20-40% meeting the diagnostic criteria for AD-type dementia as the disease progresses [20, 21]. For example, a European cohort study of 1,200 PD patients found that 27% had developed cognitive impairment severe enough to be classified as dementia, with many of these cases presenting with overlapping features of AD [5]. These statistics show the substantial proportion of individuals affected by both diseases, indicating that the overlap is not a rare occurrence, but rather a common condition, especially in elderly populations. While these findings have revealed a substantial prevalence of comorbidity between AD and PD, the likelihood of misdiagnosis could exist and lead to inaccuracies in the incidence statistics. As such, the true prevalence of comorbidity remains to be fully determined.

## 3. The Clinical manifestations

Due to the lack of unified diagnostic criteria, physicians may be unable to or may tend to avoid making a definitive diagnosis of comorbidities resulting in inconsistencies we see in epidemiological studies. While AD and PD exhibit a certain degree of symptom overlap in the late stages of the disease, attempting to separate the differences in clinical diagnosis of these comorbidities at the late stage may provide a crucial breakthrough in the development of new diagnostic standards.

The comorbidity of AD and PD was first reported by James Leverenz and Mark Sumi in 1986 [22]. The study showed that individuals diagnosed with AD may also concurrently have PD. The majority of comorbid patients tend to exhibit overlapping clinical phenotypes, particularly in the later stages of the diseases. At this stage, the disease manifestations become highly complex, and the symptoms may no longer be confined to the typical features of a single disease. Instead, they often present as overlapping with mixed clinical characteristics. AD is primarily characterized by a progressive decline in cognitive function, particularly affecting memory and other cognitive domains. Although cognitive decline is the hallmark of the disease, motor dysfunction also becomes apparent in the later stages of AD [23]. The primary manifestations of motor dysfunction in AD include apraxia [24], myoclonus [25], cautious gait and frontal gait disorder [26]. Particularly the gait alterations are often mistakenly attributed to the natural aging process and are misconstrued as being linked to muscle atrophy and decreased strength [27]. In contrast, AD patients with parkinsonism typically exhibit prominent extrapyramidal symptoms. These patients often present with rigidity, with or without tremors, but typically lack the resting tremor that is characteristic of PD [28].

PD patients typically present with motor dysfunction as they first seeking medical care. As disease progresses, patients may develop cognitive symptoms resembling those seen in AD, such as significant memory impairment and cognitive decline. These symptoms are often thought of Parkinson’s disease dementia (PDD) [29, 30]. However, patients with comorbidity are not entirely the same as those with PDD. Evidence suggests that memory impairment in PD patients with AD lesions typically involves deficits in encoding and storing information [31]. In contrast, PDD patients primarily exhibit oscillating attention. While having memory dysfunction, they typically do not experience a complete loss of encoding and storage ability [32]. Although there are certain differences between comorbid cases and AD or PD, the differences in the progression of comorbidities make it difficult to establish unified diagnostic criteria. Therefore, only through combined clinical and pathological evaluations can the occurrence of comorbidities be accurately identified.

## 4. Pathological spatiotemporal evolution of AD and PD

Given the complexity of clinical manifestations, an integrated pathological diagnosis is essential for the accurate identification and effective treatment of comorbidities. Clinical manifestations often lag behind the underlying neuropathological progression, appearing only until the pathological burdens accumulated to the point of disrupting brain function. Within this context, the spatiotemporal evolution of pathological biomarkers may serve a crucial foundation for distinguishing comorbid conditions from single entity AD or PD. Distinct disease entities exhibit characteristic patterns in terms of the initial sites of pathological protein deposition, propagation pathways, and rates of accumulation. These differences reflect fundamental divergences in pathogenic mechanisms and modes of spread within the nervous system, which may offer a potential pathological basis for differentiating comorbidities from standalone forms of AD or PD. Therefore, a comprehensive investigation into spatial and temporal distribution patterns of pathological biomarkers across brain regions and disease stages is essential—not only for improving diagnostic precision and disease classification, but also for enabling early detection, tracking disease progression, and creating personalized therapeutic strategies. Such efforts are central to advancing precision medicine in the context of neurodegenerative disorders but need more clinicopathological studies.

The classical neuropathological alterations in AD are neurofibrillary tangles (NFTs) and neuritic plaques (NPs), while in PD they are Lewy bodies (LBs) and Lewy neurites (LNs). Hyperphosphorylated Tau is a hallmark pathological feature of NFTs in AD. Tau is a member of the microtubule-associated protein family and plays a key role for neuronal and neuritic structural stability and plasticity. Hyperphosphorylation of tau leads to microtubule destabilization, disrupted protein transport and ultimately resulting in the neuronal death [33]. Aβ peptides are the primary protein component of neuritic plaques (NPs), which are formed following cleavage of the amyloid precursor protein (APP) by β-secretase and γ-secretase. Aβ1-42 is considered to be prone to self-aggregation leading to insoluble fibril deposition as amyloid plaques. In PD, α-synuclein is the primary component of LB and LN, but it is localized at synaptic terminals under normal conditions serving as a regulator for synaptic transmission, neuronal plasticity, and intracellular vesicle transport. α-Synuclein undergoes abnormal misfolding and aggregation, forming pathological aggregates such as Lewy bodies causing neurodegeneration, especially among the dopaminergic neurons in the substantia nigra.

There are significant differences in the neuropathological alterations observed in AD and PD, which develop gradually over time while maintaining a high degree of consistency across different cases [34] (Fig. 1). Braak divided NFTs, characterized by pTau and silver-stained tangle formation, into six stages: (I) occurring in the transentorhinal area; (II) spreading into the hippocampal CA1 sector; (III) to the subiculum; (IV) to the amygdala, thalamus, and claustrum; (V) to the associated isocortical areas; and (VI) to the primary sensory, motor and visual areas [35, 36]. Thal staging divides Aβ pathogenesis into five phases: (1) occurring in the isocortex; (2) spreading into the allocortex (entorhinal cortex, hippocampal formation, amygdala, and insular/cingulate cortices); (3) spreading into subcortical structures (striatum, basal forebrain, thalamus, and hypothalamus) and white matter; (4) involving the brainstem (red nucleus, substantia nigra, reticular formation, and superior and inferior colliculi); and (5) involving the pons (reticular formation, raphe nuclei, locus coeruleus) and cerebellum [37]. In PD, Braak et al. used the progressive distribution of Lewy bodies to divide PD into six stages: stages 1-2 begin in the medulla oblongata and pontine tegmentum, with lesions in the dorsal IX/X motor nucleus and/or intermediate reticular zone and later spreading to the caudal raphe nuclei, gigantocellular reticular nucleus, and coeruleus-subcoeruleus complex. Stages 3-4 involve progression to the midbrain, especially in the pars compacta of the substantia nigra. In stage 4, Lewy bodies invade the temporal mesocortex (transentorhinal region) and allocortex (CA2-plexus), while the neocortex remains unaffected.

**Figure 2.**
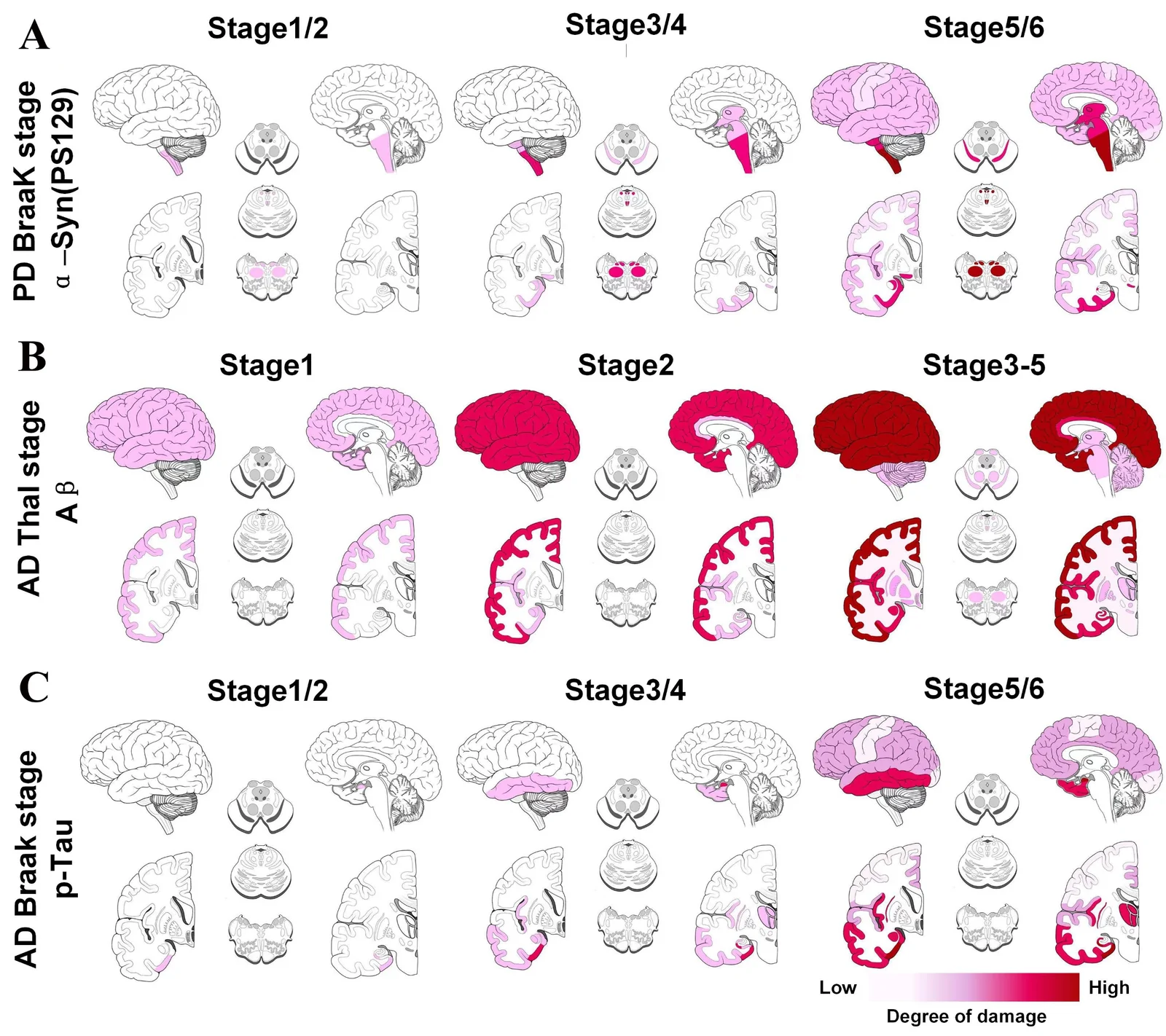
**Spatiotemporal Progression of α-syn (pS129), Aβ, and pTau Pathology in the Brain**. This figure shows the spatiotemporal progression of α-synuclein (α-syn, pS129), β-amyloid (Aβ), and phosphorylated tau (p-Tau) pathology in PD and AD, based on Braak (α-syn, p-Tau) and Thal (Aβ) staging. Brain views include lateral and medial surfaces, hippocampal/amygdalar coronal sections, and brainstem. A color gradient from pink to crimson represents increasing pathological severity. (**A**) α-syn pathology begins in stages 1-2 (medulla, pontine tegmentum, olfactory bulb; left panel, pink), extends in stages 3-4 (midbrain, basal forebrain, limbic cortex; middle panel, pink), and reaches the neocortex in stages 5-6 (right panel, pink), with earlier stages shown in magenta and crimson for reference. (**B**) Aβ pathology starts in the neocortex (stage 1; left panel, pink), progresses to the hippocampus and olfactory regions (stage 2; middle panel, pink), and finally reaches subcortical areas and cerebellum (stages 3-5; right panel, pink), with prior stages in magenta/crimson. (**C**) p-Tau pathology first affects the transentorhinal cortex and hippocampal CA1 (stages 1-2; left panel, pink), then spreads to limbic and diencephalic regions (stages 3-4; middle panel, pink), and eventually to neocortical areas (stages 5-6; right panel, pink), with earlier stages indicated similarly.

In stages 5-6, Lewy bodies spread to higher-order sensory association areas of the neocortex and the prefrontal cortex, eventually reaching first-order sensory association areas and premotor regions, with mild changes in the primary sensory and motor areas. It was shown in a number of recent studies that a significant portion of levodopa-responsive PD patients do not follow Braak’s staging system. A new staging system for Lewy body pathology has been proposed, which includes an amygdala-predominant variant and an olfactory-only stage, in addition to the traditional staging system based on the ascending course from the medulla oblongata to the cerebral neocortex [38, 39].

**Figure 3.**
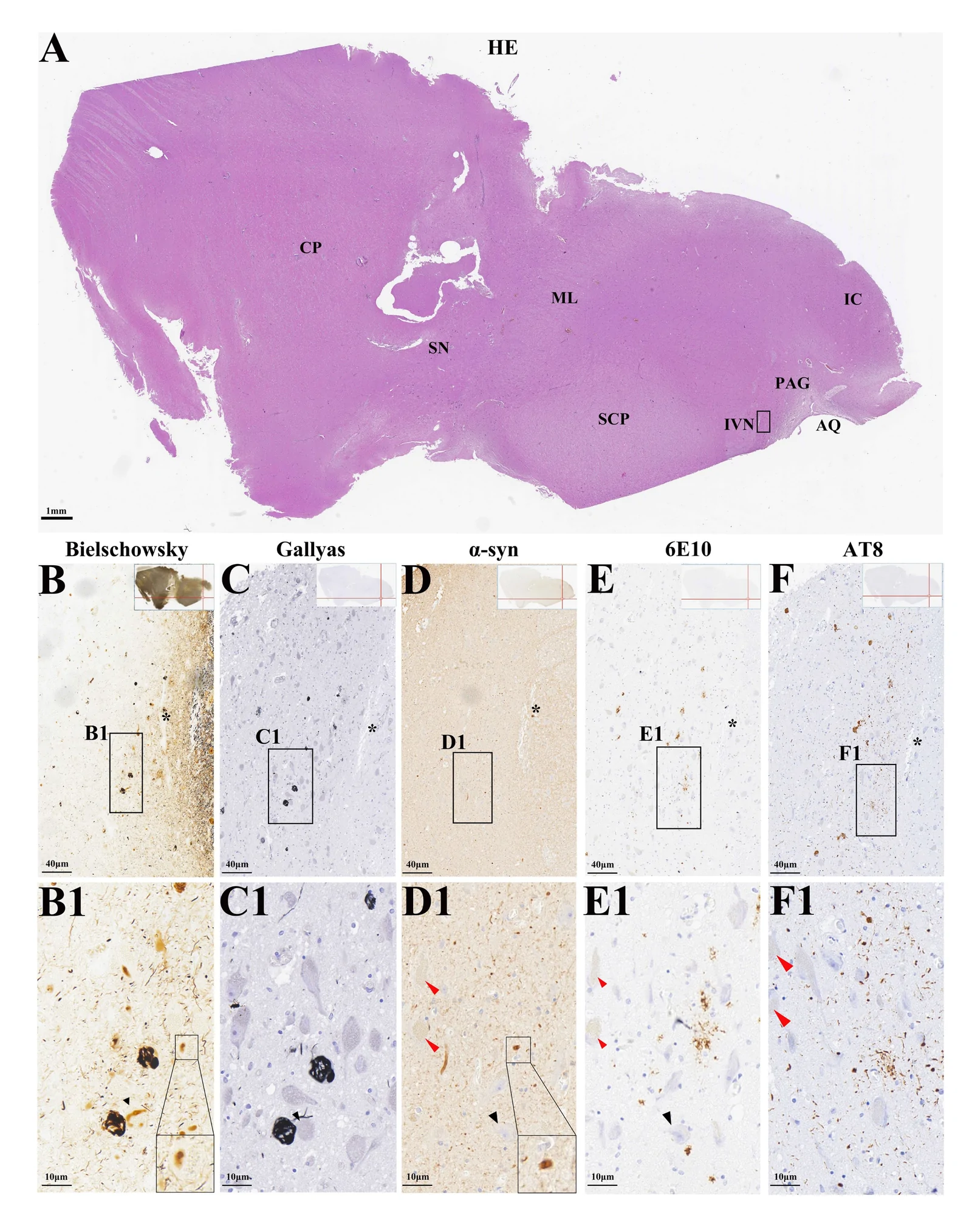
**Histopathological and immunohistochemical staining of the periaqueductal gray (PAG) at the level of the substantia nigra**. (**A**) HE stain at the substantia nigra level. (**B**) Bielschowsky silver stain of the PAG; (B1) magnified view showing ghost tangles and a Lewy body. Arrows indicate the same cell identified in adjacent sections. (**C**) Gallyas silver stain of the PAG; (C1) magnified ghost tangle. Arrows indicate the same cell as in B1. (**D**) α-Synuclein immunolabeling of the PAG; (D1) magnified view showing a Lewy body, with a further magnified inset. Black and red arrows indicate the same cell identified in adjacent sections. (**E**) 6E10 immunolabeling of the PAG; (E1) magnified view of putative Aβ deposition in a fibrillary configuration. Arrows indicate the same cell identified in adjacent sections. (**F**) AT8 immunolabeling of the PAG; (F1) magnified view showing neurofibrillary tangles and neuropil threads. Arrows indicate the same cell identified in adjacent sections. Asterisks (*) denote the same fissure observed across adjacent sections. Scale bars are indicated on each panel. Abbreviations: CP: Cerebral Peduncle, SN: Substantia Nigra, ML: Medial Lemniscus, SCP: Superior Cerebellar Peduncle, IVN: Inferior Vestibular Nucleus, PAG: Periaqueductal Gray, IC: Inferior Colliculus, AQ: Aqueduct (of Sylvius).

Different neurodegenerative diseases exhibit distinct patterns of spatiotemporal neuropathological progression. However, the brains of patients with comorbidity often exhibit an accumulation of multiple pathological hallmarks. Studies suggest that alongside senile plaques and neurofibrillary tangles, Lewy body lesions and α- Syn pathology are also present in the brains of patients who have been clinically diagnosed with AD during life, as revealed by postmortem examination [40]. Similarly, other studies have also reported that patients diagnosed antemortem with PD, in addition to exhibiting α-syn pathology and nigral degeneration, also display extensive neurofibrillary tangles and amyloid plaques upon postmortem pathological analysis [41]. It is clear that the brain regions affected in the early stages of AD and PD are different. However, as the diseases progress, pathological changes spread to some of the same brain regions.

We performed serial section analysis of the aforementioned AD and PD comorbid case in the midbrain sections, using HE (Fig. 3A), Bielschowsky silver (Fig. 3B) and Gallyas silver (Fig. 3C) stains, and immunohistochemical labeling for α-syn (Fig. 3D), 6E10 (Fig. 3E), and AT8 (Fig. 3F). By identifying the same cells across adjacent sections (indicated by arrows), we observed co-localization of multiple pathological markers within the periaqueductal gray (PAG) region. Bielschowsky stain revealed prominent ghost tangles and neuropil threads (Fig. 3B, B1), along with the presence of Lewy bodies (Fig. 3B1, enlarged image). Gallyas stain confirmed the presence of ghost tangles and neuropil threads (Fig. 3C, C1). α-Synuclein IHC identified both Lewy neurites (LNs) and Lewy bodies (LBs) (Fig. 3D, D1, and enlarged image). 6E10 immunolabeling showed diffuse amyloid plaques and swollen axons (Fig. 3E, E1), while AT8 immunolabeling further confirmed the presence of tangles and neuropil threads (Fig. 3F, F1).

During the progression of comorbid neurodegenerative conditions, the brain regions affected by different pathological markers may vary due to differences in their respective onset times and disease courses. However, regardless of whether AD or PD manifests first, both may eventually impact overlapping brain regions at certain stages of disease progression. There are also instances in which pathological changes remain relatively independent and do not converge within the same anatomical areas. This complex and heterogeneous pathological pattern is reflected by the high degree of individual variability and dynamic evolutionary characteristics of neurodegenerative diseases. The spatial and temporal mismatch in the accumulation of multiple pathological proteins—such as Aβ, pTau, and α-synuclein—may exacerbate neuronal dysfunction in a nonlinear manner, thereby influencing both clinical manifestations and therapeutic responses [34, 42].

## 5. Similarity of propagation of tau, α-syn and Aβ

The pathological process of comorbidity is not static but may follow a certain spatiotemporal pattern of progression. The deposition of pathological proteins typically originates in specific brain regions and gradually spreads to other areas as the disease advances. This progression is not random and tends to follow a predictable propagation trajectory, suggesting the presence of a shared underlying biological mechanism. Studies have suggested that abnormal proteins such as Aβ, pTau and α-synuclein can propagate trans-synaptically between neurons through a "seeding" mechanism, forming a chain-like accumulation process that drives progressive structural and functional deterioration of the brain [35, 43-47].

Tau and α-synuclein may spread between synapses and cells via exosomes and extracellular vehicles (EVs). Tau and α-synuclein are detected in the interstitial fluid in mouse and human brains, implicating that these proteins, secreted into the extracellular space, may mediate cell-to-cell transmission of pathology [48-50]. Pathological Tau and α-synuclein can form exosomes and be released into the extracellular milieu by exocytosis [51-53]. Upon secretion into the extracellular space, these proteins are internalized by other neurons through an endocytic mechanism [44, 54-56]. A recent study suggests Tau and α-synuclein fibrils engage with heparan sulfate proteoglycans (HSPG) on the cell membrane, which are then internalized through micropinocytosis [54]. A further study revealed that α-synuclein fibrils are recognized by the lymphocyte-activation gene 3 (LAG3) receptor on the cell surface, facilitating their internalization through receptor-mediated endocytosis [56]. Upon internalized, Tau and α-synuclein fibrils are directed towards the endolysosomal pathway, where they undergo partial degradation in lysosomal compartments [57, 58]. If lysosomal integrity is compromised, these fibrils are released into the cytoplasm, where they may seed the aggregation of monomeric Tau and α-synuclein. Following exosome uptake these external vesicles may be incorporated into endogenous endosomal structures and transported along axons, enabling the transfer of pathological proteins between neurons [59]. However, the mechanisms regarding endocytosis, exocytosis, intra- and inter-neuronal transmission *in vivo*, especially in the human brain, remain to be established.

Aβ is also believed to propagate in a prion-like pattern, although its propagation mechanism differs somewhat from that of Tau and α-synuclein. Aβ may spread via the diffusion or transport of Aβ seeds (tissue propagons), or through indirect effects (non-prion-like) [60]. Recent studies suggest that the intracellular accumulation of Aβ disrupts the homeostatic control of Aβ levels [61]. The intercellular accumulation of Aβ acts like a seed spreading in a permissive environment. Once the homeostatic balance of Aβ levels is disturbed, Aβ deposition begins to develop in the affected regions. Some animal experiments support this concept, as when Aβ seeds are injected into the striatum, a brain region where spontaneous Aβ diffusion and deposition are typically absent or rare, it results in the development of diffuse Aβ deposits during the observation period [62].

This "seeding and propagation" mechanism of pathological propagation not only deepens the understanding of the spatiotemporal evolution of disease but also provides a mechanistic explanation for the co-existence and synergistic interaction of multiple pathological proteins observed in comorbid conditions. When each protein undergoes misfold, aberrantly accumulate, it can spread the “seed” to adjacent neurons through a “seed and spread” process. When Aβ, pTau, and α-synuclein are present within the same or neighboring brain regions, cross-seeding among them may lead to a cascade where the misfolding of one protein promotes the misfolding and aggregation of the others. In patients with concurrent AD and PD, Aβ, pTau, and α-synuclein tend to accumulate persistently in their respective target regions, presenting a co-distribution pattern. Such cross-seeding intensifies disruption in neural circuits and leads to more complex, heterogeneous clinical manifestations as well as a more rapid disease progression. Moreover, the interplay among multiple pathological proteins may lead to more intricate pathological burdens and exacerbated impairment of brain function. Therefore, elucidating the propagation dynamics and interaction mechanisms of distinct pathological proteins is essential for improving early detection, risk assessment, and the development of targeted therapeutic strategies in comorbidity.

## 6. Interaction between Tau and α-syn, Aβ and α-syn

In comorbid states, multiple pathological proteins tend not to spread independently but rather exhibit spatial co-localization and functional convergence across several key brain regions. These proteins may engage in complex interactions during their propagation. Evidence suggests that Aβ, pTau, and α-synuclein may interact at the molecular level, for instance, by co-activating neuroinflammatory pathways, modulating cellular stress responses, or directly promoting each other’s misfolding and aggregation [63, 64]. Such interactions may not only accelerate the spatial spread of pathological burden but also lead to more complex pathological accumulation and more severe impairment of brain function, thereby providing a pathological basis for the heightened clinical heterogeneity observed in comorbid patients.

Tau plays a crucial role in stabilizing and promoting microtubule polymerization within neuronal cell bodies and processes [33, 65], while α-synuclein regulates synaptic function [66]. Aβ is generated by the enzymatic cleavage of APP and is expressed in the membrane systems [67]. Although Tau and α-synuclein aggregation are hallmark features of different neurodegenerative diseases, emerging evidence suggests that there is a close connection between these two pathological proteins [68-71]. A study of a rare familial case of Parkinson's disease with the A53T α-synuclein mutation revealed inclusions of both α-synuclein and Tau [72, 73]. In sporadic PD, the coexistence of Tau pathology and α-synuclein aggregation is also reported [74-76]. Similarly, Aβ was reported to co-exist with α-synuclein [77-80]. In the neocortex of a patient diagnosed with the Lewy body variant of Alzheimer's disease, α-synuclein was localized in a pattern encircling amyloid plaques [80]. In the PD patient with AD lesions, α-synuclein aggregation was co-localized with Aβ deposition [81]. These lines of evidence suggest a potential interaction between Aβ and α-synuclein.

α-Synuclein and Tau may interact with each other, influencing their aggregation. In vitro studies have shown that overexpressed α-synuclein can promote the phosphorylation of Tau at S262 and S356 through protein kinase A (PKA) [82-85]. Moreover, α-synuclein could also promote the phosphorylation of Tau via increased glycogen synthase kinase 3β (GSK3β) activity and the formation of a GSK3β-α-synuclein-Tau complex [83, 86]. GSK3β can phosphorylate a majority of sites on Tau, resulting in the formation of paired helical filaments and neurofibrillary tangles [87-89]. It has been shown that phosphorylated Tau (pTau) can increase the phosphorylation of α-synuclein and enhance α-synuclein aggregation [68]. Animal studies have reported that Tau aggregation regulates synuclein pathology but does not directly affect the spreading of α-synuclein in the brain [90]. Furthermore, coincubation of Tau and α-synuclein synergistically promotes the fibrillization of both proteins in vitro [70]. Tau has been detected as a ligand of α-synuclein through affinity chromatography, suggesting a physical interaction between the two proteins [71]. In vivo, the inoculation of pathological conformers of α-synuclein and Tau into healthy tissue has been shown to cross-seed α-synuclein or Tau aggregation [91]. Furthermore, when α-synuclein and Tau were overexpressed using viral vectors, the co-occurrence of Tau and α-synuclein pathology was still observed. In rats, stereotaxic injection of lentivirus to overexpress α-synuclein led to increased levels of pTau. Similarly, rats transduced with Tau and mutant P301L Tau showed an increase in both α-synuclein and phosphorylated α-synuclein levels [92]. These data suggest significant physical interaction between Tau and α-synuclein. Alternatively, α-synuclein and Tau may interact indirectly through their impact on neuronal physiology, such as activating kinases, modulating excitability, and influencing gene expression, or by causing neuronal stress/injury and activating host responses.

**Figure 4.**
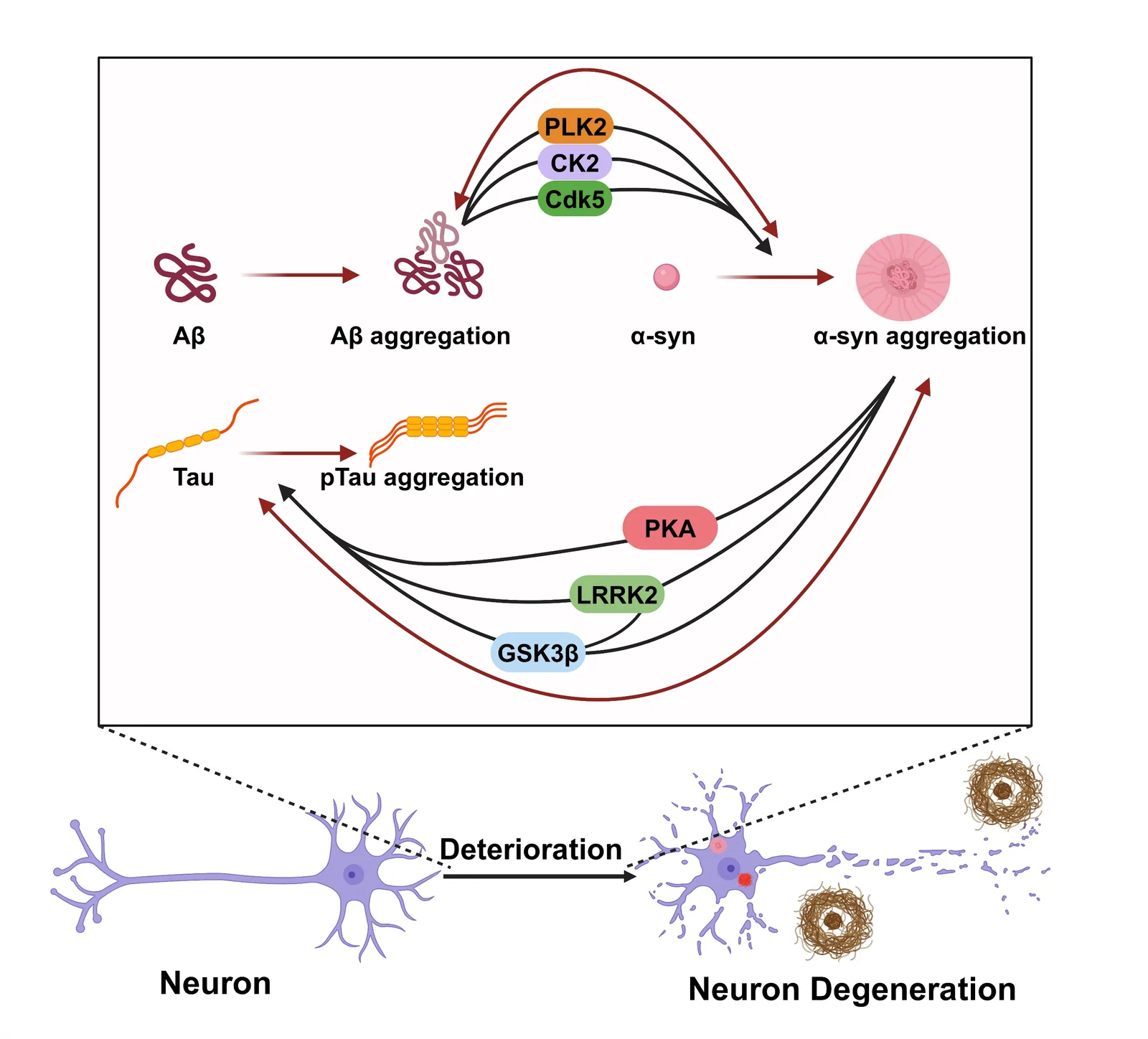
**Potential pathological protein interactions promoting AD and PD comorbidity**. In the brains of patients with comorbid AD and PD, complex interactions msyt exist between key pathological proteins, which accelerates neuronal degeneration. In comorbid patients, Aβ and Tau can each directly interact with α-syn, forming a vicious cycle (Red arrow). Specifically, Aβ and pTau aggregates can promote the misfolding and aggregation of α-syn, while α-syn aggregates can, in turn, enhance the phosphorylation and aggregation of pTau, further exacerbating neurotoxicity. In addition to these direct interactions, there are also complex indirect regulatory mechanisms among Aβ, pTau, and α-syn. Aβ aggregation can indirectly promote the formation, misfolding and aggregation of α-syn through PLK2 (Polo-like kinase 2), CK2 (Casein kinase 2), and Cdk5 (Cyclin-dependent kinase 5). Meanwhile, under the regulation of PKA (Protein kinase A), LRRK2 (Leucine-rich repeat kinase 2), and GSK3β (Glycogen synthase kinase 3 beta), the interaction between pTau and α-syn is further enhanced, mutually promoting each other’s phosphorylation, misfolding, and aggregation (Black arrow). These direct and indirect molecular interactions ultimately lead to neuronal dysfunction, accelerated neurodegeneration, and the progression of neurodegenerative diseases.

Aβ also interacts with α-synuclein to form complexes [93]. In normal neurons, α-synuclein exists in a random coil conformation as it is transported to the synaptic terminal, where it assembles on the synaptic vesicle surface, adopting a stable α-helical structure. Under normal conditions, cytoplasmic α-synuclein does not interact with Aβ peptides embedded within the pre-synaptic membrane. However, under pathological conditions, abnormal accumulation of free cytoplasmic α-synuclein at the pre-synaptic membrane may occur due to impaired axonal transport, defective proteolysis, a reduced number of synaptic vesicles, or other contributing factors. As a result, cytosolic α-synuclein in its random coil form transforms into a membrane-bound α-helical structure. This membrane-bound α-synuclein comes into contact with membrane-associated Aβ peptides, potentially initiating pathogenic interactions. Other studies have suggested that the PI3K pathway is the reason why Aβ1-42 interacts with α-synuclein. The evidence of in vitro experiment suggests that both Aβ and α-synuclein could activate the PI3K pathway [94, 95]. The PI3K inhibitor, LY294002 could block Aβ and α-synuclein-dependent elevation in α-synuclein and Aβ, respectively [79]. Some studies also suggest that the indirect interaction between Aβ and α-synuclein is mediated through Aβ-induced phosphorylation of α-synuclein by several kinases, such as Cdk5, casein kinase 2, and polo-like kinase 2 (PLK2) [80, 96]. These results show that there are interactions between Tau and α-synuclein, Aβ and α-synuclein, which further exacerbates neuronal damage (Fig. 4), potentially serving an important mechanism for the accelerated deterioration of neurodegenerative diseases in the comorbidity of AD and PD.

## 7. Conclusions and future directions

In the current review, we summarize the epidemiological characteristics, clinical manifestations, pathological progression, and potential molecular mechanisms associated with the comorbidity of AD and PD, emphasizing its importance as a critical and insufficiently acknowledged issue in both clinical practice and research. In clinical practice, the comorbidity of AD and PD is frequently misdiagnosed in the advanced stages of either AD or PD, hindering prompt identification and effective clinical management. With advances in the detection of the various pathological biomarkers Aβ, pTau, and α-synuclein, increasing attention has been directed toward integrating their spatiotemporal distribution patterns with the clinical phenotypes of patient to improve diagnostic precision and support the development of multi-targeted therapeutic strategies.

At the clinic-level, patients with comorbidity exhibit a wide spectrum of complex and heterogeneous symptoms that differ from those observed in pure AD or PD cases. The emergence of motor symptoms (such as rigidity and tremors) in late-stage AD, or the onset of cognitive impairment in the advanced stages PD, is closely related to the accumulation and spread of pathological markers as the disease progresses to corresponding functional brain regions. However, in patients with comorbidity, motor symptoms and cognitive impairment often emerge concurrently or sequentially at early stage of the disease, rather than simply reflecting a summation of late-stage features of AD and PD. For instance, motor symptoms may lack the classic tremor-dominant presentation commonly seen in PD, instead manifesting as atypical gait instability or axial motor deficits [97]. Likewise, cognitive impairment may deviate from the typical amnestic profile observed in AD, with a greater prominence of attentional fluctuations, visuospatial dysfunction, or executive deficits [98, 99]. The clinical pattern—characterized by asymmetry in symptom severity and atypical combinations—suggests that the temporal sequence and propagation of pathological protein accumulation in comorbid states may differ from those seen in isolated AD or PD, thereby indicating a potentially distinct underlying pathological mechanism.

The clinical heterogeneity described above may, to a large extent, be attributed to differences in the spatiotemporal progression, regional distribution, and aggregation patterns of pathological proteins such as Aβ, pTau, and α-synuclein within the brain. For example, in patients with AD, the abnormal aggregation of Aβ and pTau in the midbrain and basal ganglia can impair motor coordination and regulation, leading to tremors and other motor dysfunctions. In patients with PD, the aggregation and deposition of α-synuclein in the hippocampal region contributes to neuronal damage and loss, which subsequently leads to cognitive impairment. Notably, even when AD pathology extends into the midbrain or medulla, or when PD pathology spreads to the limbic system (such as the hippocampus or amygdala), the resulting pathological features often retain the hallmark characteristics of their respective primary diseases. In other words, although these regions are not traditionally regarded as “classically affected areas”, their pathological expressions remain highly disease-specific. However, in patients with comorbidity, even the distribution of Aβ and α-synuclein exceeds the boundaries of their typical target regions, their classically affected areas still exhibit hallmark pathological features of the primary disorders. Moreover, certain critical brain regions may present with co-accumulation of multiple pathological markers, forming what may be termed a "mixed" pathological state. As demonstrated in Figure 3 of this study, the periaqueductal gray (PAG) exhibits simultaneous deposition of Aβ plaques, neurofibrillary tangles, and neuritic plaques (NPs), along with the coexistence of Lewy bodies (LBs) and Lewy neurites (LNs). Evidence suggests that the coexistence of multiple pathological proteins is not merely coincidental but may reflect specific mechanistic synergism [100]. Aβ, pTau and α-synuclein all exhibit prion-like “seed and spread” model and are highly prone to cross-seeding mechanisms, whereby the misfolding and aggregation of one pathological protein can serve as a “seed” to induce the aggregation of others [101]. Studies have shown that Aβ deposition can trigger tau aggregation and abnormal α-synuclein aggregation may also accelerate the aggregation of both Aβ and Tau [68, 91]. This molecular-level “mutual seeding” implies that once the aggregation of one pathological protein is initiated, it may propagate through template replication and conformational transmission, thereby inducing the misfolding of others and accelerating pathological progression. At the same time, evidence indicates that pathological proteins propagate trans-synaptically along anatomically connected brain regions with spreading closely aligned with the underlying architecture of neural networks [102]. Particularly in the context of coexisting proteinopathies, the propagation pathway of Aβ, pTau and α-synuclein may overlap or converge in specific brain regions, forming so-called “pathological convergence zones”. These convergence zones provide a spatial substrate for cross-seeding, thereby further reinforcing the cooperative and mutually enhancing aggregation of pathological proteins. Thus, pathological propagation is not an isolated process but a dynamic “interactive propagation” phenomenon, in which cross-seeding at the molecular level and convergence of spreading pathways at the systems level form a positive feedback loop that drives pathological aggregation and dissemination. Furthermore, such pathological crosstalk may be mediated by multiple mechanisms, including cross-seeding, co-activation of neuroinflammatory responses, competition or overlap within degradation pathways such as the ubiquitin-proteasome system and the autophagy-lysosomal system, or self-repairing or regenerative responses of neuronal and synaptic elements. The emergence of these “pathological convergence zones” not only complicates the clinical phenotype of comorbid patients but also poses new challenges for early diagnosis and the development of targeted therapeutic strategies.

Therefore, the distinct clinical manifestations and complex pathological features of comorbidity of AD and PD further underscore the importance of achieving precise diagnosis and individualized treatment. Compared to single-pathology cases, patients with comorbidity often exhibit atypical combinations of cognitive, motor, and neuropsychiatric symptoms across multiple domains, offering a novel "phenotypic window" for early clinical identification. In particular, the coexistence of multiple pathological markers within specific brain regions may serve as a key indicator of comorbid pathology. Therefore, integrating clinical phenotypes with pathological imaging data to establish more sensitive and specific diagnostic criteria has become a central focus of current research.

In recent years, with the continuous advancement of molecular imaging technologies, in vivo imaging modalities, represented by positron emission tomography (PET-CT), have played an increasingly important role in the diagnosis of neurodegenerative diseases. By employing radiotracers targeting specific pathological proteins (such as [¹¹C] PIB for Aβ, [¹^8^F] Flortaucipir for pTau, and peptide-based probes for α-synuclein), researchers have begun to achieve in vivo visualization of multiple proteinopathies [103-107]. These imaging tools not only provide insights into the spatial distribution patterns and aggregation densities of these pathological proteins in the brain, but also enable the capture of their spatiotemporal dynamics through longitudinal imaging. Compared to traditional diagnostic approaches based on clinical symptoms or structural imaging, molecular imaging offers a more direct and earlier reflection of pathological progression, thereby providing critical support for elucidating the mechanisms of interaction between coexisting pathologies such as AD and PD. In addition to imaging technologies, the analysis of the biomarker in cerebrospinal fluid (CSF) has also become an increasingly important tool for assessing pathological burden. Measuring the concentrations of Aβ, pTau, and α-synuclein in CSF, may indirectly infer their aggregation patterns and pathological states within the brain. This fluid-based biomarker approach is particularly valuable in clinical scenarios where imaging coverage is limited or cost-prohibitive, offering a feasible alternative for screening and subclassifying neurodegenerative diseases. Therefore, with the advancement and integration of multi-modal techniques, the combined monitoring and dynamic tracking of multiple pathological proteins is likely to become a critical breakthrough in understanding the pathogenesis and progression of mixed neurodegenerative conditions, such as comorbid AD and PD.

Moreover, in the context of personalized treatment, identifying the interactions among different pathological proteins and understanding their spatiotemporal evolution across various brain regions can facilitate the development of multi-targeted intervention strategies. For instance, for the patients exhibiting significant co-expression of α-synuclein and pTau, a combination of anti-Tau and anti-α-synuclein immunotherapies may help suppress the aggregation of multiple protein species. In the future, as protein-specific tracers, imaging analysis algorithms, and multi-omics diagnostic platforms continue to improve, the precision of comorbidity detection and pathological subtyping is expected to increase. This will further advance individualized therapeutic approaches centered on specific proteinopathy profiles.
